# Thermomechanical Properties of Zeta (Ag_3_In) Phase

**DOI:** 10.3390/ma16227115

**Published:** 2023-11-10

**Authors:** Xunda Liu, Hiroaki Tatsumi, Zhi Jin, Zhong Chen, Hiroshi Nishikawa

**Affiliations:** 1 Joining and Welding Research Institute, Osaka University, Ibaraki 567-0047, Osaka, Japan; tatsumi.jwri@osaka-u.ac.jp (H.T.); jinzhi711@gmail.com (Z.J.); 2 Graduate School of Engineering, Osaka University, Suita 565-0871, Osaka, Japan; 3 School of Materials Science and Engineering, Nanyang Technological University, 50 Nanyang Avenue, Singapore 639798, Singapore; aszchen@ntu.edu.sg

**Keywords:** intermetallic compounds, transient liquid phase bonding, die-attach materials, thermomechanical properties, nanoindentation

## Abstract

The thermomechanical properties of materials within die-attach joints play an essential role in assessing the reliability of high-power modules. Ag-In transient liquid phase (TLP) bonding serves as an alternative method for die attachment. However, relevant material data for the ζ (Ag_3_In) phase, one of the Ag-In intermetallic compound (IMC) products of TLP bonding, are limited. This paper proposes an approach to fabricate a densified and pure bulk sample of the ζ (Ag_3_In) phase. The thermomechanical properties of the ζ (Ag_3_In) phase were subsequently investigated at elevated temperatures and compared to those of other IMCs frequently observed in die-attach joints. As the temperature increased from 30 °C to 200 °C, the hardness of the ζ (Ag_3_In) phase decreased linearly from 1.78 GPa to 1.46 GPa. Similarly, the Young’s modulus also decreased linearly from 82.3 GPa to 66.5 GPa. These properties rank among the lowest levels compared to those of other IMCs. The average coefficient of thermal expansion within the temperature range of 70 °C to 250 °C was approximately 18.63 ± 0.61 μm/m/°C, placing the ζ (Ag_3_In) phase at a moderate level. When considering its potential for mitigating thermal stress, these combined properties render the ζ (Ag_3_In) phase an appropriate material choice for die-attach joints compared to other IMCs.

## 1. Introduction

High-power modules play a pivotal role in regulating electrical power during storage, conversion, and distribution processes [1,2]. With increasing environmental awareness, the utilization of cleaner and more sustainable energy sources, such as wind, water, and solar energy, has gained widespread recognition [3]. Consequently, the increasing demand for efficient energy conversion and transportation has imposed stringent requirements on the reliability of high-power modules. Unfortunately, conventional silicon-based high-power modules are approaching their theoretical limits because of their inherent silicon chip properties, rendering them inadequate to meet these stringent demands [4]. In response to these challenges, wide-band-gap (WBG) semiconductor materials have emerged as promising alternatives to replace silicon in high-power modules, owing to their exceptional properties, including their high breakdown voltage, low switching losses, and high-temperature tolerability [4]. The WBG-based chips can operate with greater energy conversion efficiency under higher power densities and extreme temperature conditions, making them more suitable for the aforementioned applications [5]. Despite the notable performance improvements achievable with WBG-based chips, the accompanying die attachment techniques, which involve a connection between the chip and the substrate, have not yet been well developed [6]. Conventional die attachment materials, such as Pb-free solders, cannot withstand the high-temperature working conditions of WBG chips because of their low melting temperatures [7]. Therefore, developing novel die attachment processes and materials, along with investigating their thermomechanical properties to assess their joint reliability, is necessary.

A promising approach for die attachment is transient liquid phase (TLP) bonding, which can be conducted at low temperatures while forming a joint with a high melting temperature [8]. TLP bonding occurs through the interdiffusion between a molten alloy with a lower melting temperature and a metal substrate, resulting in the formation of an intermetallic compound (IMC) with a higher melting temperature [8]. A high-temperature-resistant die attachment is achieved once the IMC formation consumes all the molten alloy and occupies the entire die-attach joint. TLP bonding offers advantages such as facile processing and cost-effectiveness and has attracted considerable attention and extensive research in recent years. Numerous material combinations have been explored for TLP bonding, including the use of Sn, solder, In, and Ga as the molten layers and Cu, Ag, Ni, and Au as substrates [9,10,11,12,13,14,15,16]. Among these, the Ag-In combination has shown promising potential for TLP bonding. Our previous research demonstrated that Ag-In interdiffusion exhibits high reaction kinetics [17], leading to reduced bonding times [18]. Additionally, good performance of Ag-In IMC joints in thermal cycling reliability tests has been reported [19,20]. However, to date, the understanding of Ag-In IMCs remains limited, and their performance as die-attach joints remains uncertain, owing to a lack of information on their essential properties, particularly their thermomechanical properties.

The thermomechanical properties of the joint material are critical because they enable the assessment of thermal stress that occurs among WBG-based chips, die-attach joints, and substrates during operation [21]. This serves as a vital indicator for predicting the lifetime of die attachments. For example, Chen C.T. et al. have investigated the Young’s modulus, shear modulus, and coefficient of thermal expansion (CTE) of sintered Ag, another promising material for die attachment, at elevated temperatures [22]. By employing a finite element modelling simulation with the acquired materials data, they successfully evaluated the performance of sintered Ag joints under thermal shock conditions and proposed an optimized joining structure for die attachment [23]. For the same purpose, in terms of TLP bonding, Chen Z.W et al. investigated the Young’s modulus and creep rate of Cu_6_Sn_5_ at elevated temperatures to evaluate the fatigue life of the die-attach joints [24]. Despite the critical importance of thermomechanical properties in die attachment, research on IMCs in this context remains limited. The primary reason for this lies in the difficulties in fabricating bulk and pure IMC samples for measurements. Consequently, to utilize Ag-In TLP bonding for die attachment, fabricating a bulk and pure IMC sample as formed in the die-attach joint and investigating their thermomechanical properties is imperative. Our previous study identified two types of IMCs, Ag_9_In_4_ and the ζ (Ag_3_In) phase, in Ag-In TLP-bonded joints [25]. The Ag_9_In_4_ joints exhibited shear strengths of up to 53 MPa, whereas the ζ (Ag_3_In) joints exhibited shear strengths of up to 82.9 MPa. Despite the slight difference in Ag composition, approximately 3–4 at. %, they exhibited significant variations in shear strength, fracture mechanisms, and hardness. Although the fabrication and thermomechanical properties of Ag_9_In_4_ were systematically studied in our previous study [26], those of the ζ (Ag_3_In) phase have remained unreported, generating considerable interest. The ζ (Ag_3_In) phase has demonstrated superior results in shear tests; therefore, to assess its suitability as a die-attach joint, fabricating a bulk sample of the ζ (Ag_3_In) phase and determining its thermomechanical properties is essential.

In this study, the thermomechanical properties of the ζ (Ag_3_In) phase, including its hardness, Young’s modulus, and CTE, were investigated at elevated temperatures. Building on our previous research, in which we summarized the influence of fabrication processes on the porosity and purity of Ag_9_In_4_ [26], we successfully produced dense and pure bulk ζ (Ag_3_In) phase samples via casting and heat treatment. Subsequently, the hardness and Young’s modulus of the ζ (Ag_3_In) phase at elevated temperatures ranging from 30 °C to 200 °C were investigated using nanoindentation tests. Furthermore, the average CTE value across a temperature range of 70 °C to 250 °C was determined. For comparison, we referenced the corresponding properties of IMCs commonly used in die-attached joints, including Cu_6_Sn, Cu_3_Sn, Ni_3_Sn_4_, Ag_3_Sn, and Ag_9_In_4_. The thermomechanical properties of the ζ (Ag_3_In) phase, as determined in this study, are reported for the first time and constitute a valuable addition to material databases for assessing the performance of joint layers in high-power modules.

## 2. Materials and Methods

**Figure 1:**
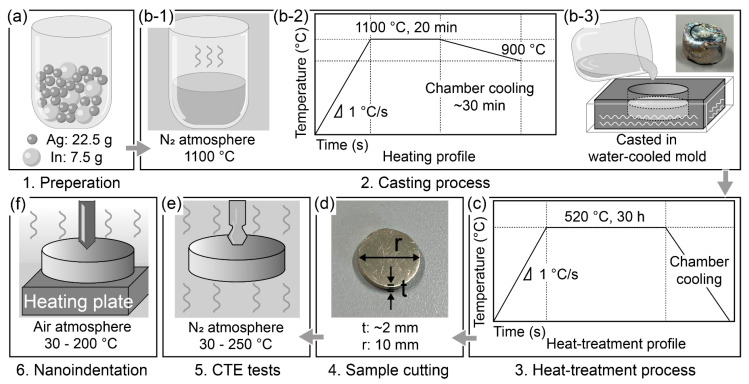
Schematics of experimental procedures. (**a**) Raw material preparation. Casting process, with (**b-1**) depicting melting conditions, (**b-2**) showing temperature profiles during melting, and (**b-3**) illustrating the casting process in the water-cooled mold. (**c**) Temperature profile during the heat treatment process. (**d**) Dimensions of samples for measurements. (**e**) CTE tests. (**f**) Nanoindentation tests.

The production of a densified and pure ζ (Ag_3_In) phase is a prerequisite for measuring its thermomechanical properties. As mentioned earlier, in a previous study, we systematically investigated the fabrication process and successfully fabricated densified and pure Ag_9_In_4_ (further details are available in the supplementary information in [26]). The conclusions obtained were referenced when the ζ (Ag_3_In) phase was fabricated in this study, and the following experimental procedures were proposed, as schematically outlined in Figure 1.

**Figure 2:**
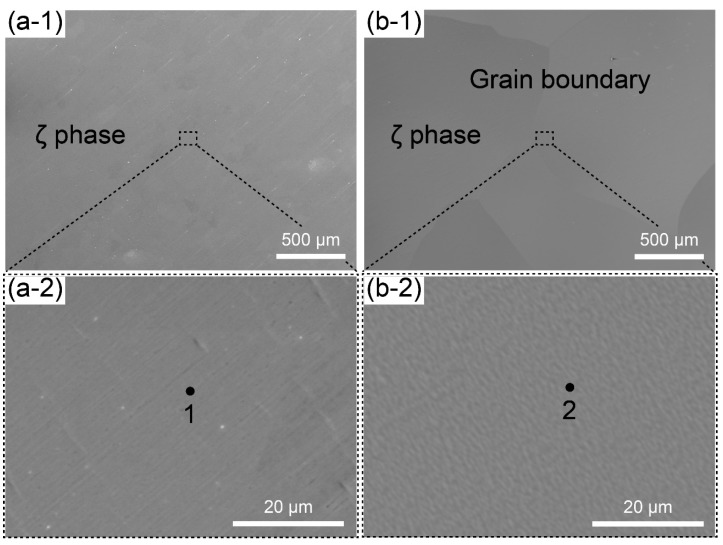
SEM images of (**a-1**,**a-2**) as-solidified ingot and (**b-1**,**b-2**) heat-treated sample.

**Figure 3:**
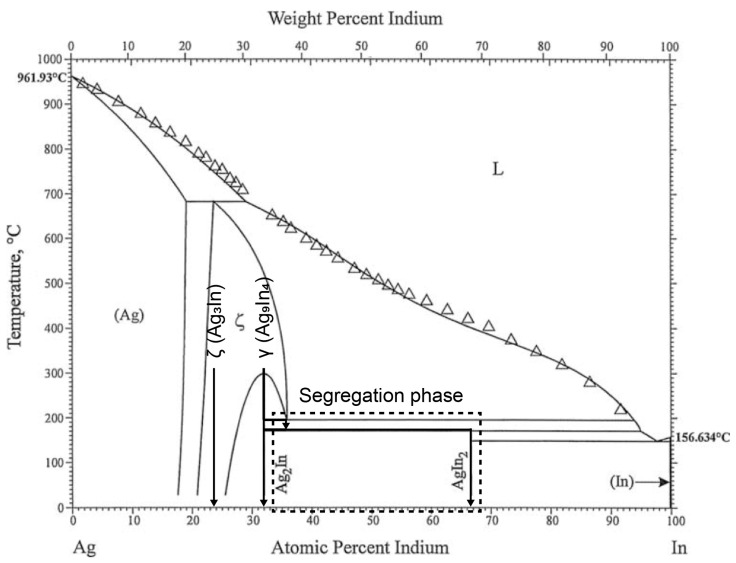
Ag-In phase diagram [28].

First, pure Ag (99.99%) and pure In particles (99.99%) of diameters of 1 and 2–3 mm (Nilaco Ltd., Tokyo, Japan), respectively, were used as raw materials. Following the atomic ratio of the ζ (Ag_3_In) phase derived from Ag-In TLP-bonded joints [25], 22.5 g of Ag and 7.5 g of In were measured and mixed in a crucible, as illustrated in Figure 1a. To obtain the Ag-In alloy ingot, the casting process was performed using the pre-measured Ag and In mixture, as shown in Figure 1(b-1,b-2,b-3). The mixed samples were placed in a resistance furnace and heated to 1100 °C for 20 min to melt, as shown in Figure 1(b-1). Subsequently, the samples were cooled in the chamber until they reached 900 °C, at which point they were removed. The temperature profile during the melting process is shown in Figure 1(b-2). An inert nitrogen atmosphere was maintained in the furnace to prevent oxidation. The molten alloy was then poured into a cavity within a water-cooled steel mold, measuring 10 mm in diameter and 15 mm in depth, as shown in Figure 1(b-3). This process yielded an as-solidified ingot with a diameter of 10 mm, as also shown in Figure 1(b-3). Subsequently, the solidified ingots underwent heat treatment in a vacuum-resistant furnace. Specifically, they were subjected to a temperature of 520 °C for 30 h to achieve homogenization. The heat-treated samples were then cooled to room temperature in the chamber. The temperature profile during the heat treatment process is shown in Figure 1c.

The heat-treated samples were cut into cylindrical shapes, approximately 2 mm in thickness and 10 mm in radius, as shown in Figure 1d. These specimens were prepared for subsequent high-temperature nanoindentation tests and CTE measurements. The samples cut for observations were ground using 400–4000 grit SiC papers and polished with 1 μm alumina slurries. The microstructures and compositions were characterized using scanning electron microscopy (SEM; JSM-IT200, JEOL Ltd., Tokyo, Japan) and energy-dispersive spectroscopy (EDS; JED-2300, JEOL Ltd., Tokyo, Japan). The phases were characterized using X-ray diffraction (XRD; Ultima IV, Rigaku, Tokyo, Japan). The samples cut for nanoindentation tests were further polished using 1–0.05 μm alumina slurries. A Berkovich diamond indenter was used, and a constant loading method was applied. The nanoindentation tests (ENT-5, Elonix Ltd., Tokyo, Japan) were performed under a peak load of 20 mN with a dwell time of 30 s at temperatures of 30 °C, 50 °C, 75 °C, 100 °C, 125 °C, 150 °C, 175 °C, and 200 °C, respectively. The Young’s modulus and hardness were determined at each temperature using the unloading method proposed by Oliver and Pharr [27]. Ten measurements were conducted at each temperature to obtain average values. Following testing, a 20 s hold at 2 mN was applied to correct for thermal drift. The samples cut for the CTE measurements were finished by grinding with 4000 grit SiC paper. The CTE was examined using thermal analysis equipment (TMA-7100, Hitachi Ltd., Tokyo, Japan) within a temperature range of 30 °C to 250 °C. Three measurements were obtained for the two specimens to determine the average CTE values.

## 3. Results

### 3.1. Characterization of Bulk ζ (Ag_3_In) Samples

The SEM images of the as-solidified ingots and heat-treated samples are shown in Figure 2(a-1,a-2,b-1,b-2), respectively. No segregation phases can be observed in either samples. This differs from the as-solidified ingot when fabricating Ag_9_In_4_, where In-rich segregation phases are prevalent [26]. The key difference is that the ingots with the 3Ag:In composition did not undergo phase transitions during cooling, as indicated by the phase diagram in Figure 3. In contrast, with Ag_9_In_4_, even a slight segregation caused by solidification was likely to promote peritectic transformation at 205 °C and 166 °C, leading to the formation of In-rich segregation phases. Consequently, the fabrication of a pure sample of the ζ (Ag_3_In) phase was simpler than that of pure Ag_9_In_4_. Following the heat treatment, as shown in Figure 2(b-1), the grains grew to the millimeter scale. This ensured the consistency of the nanoindentation results, as the tests could be conducted within a single grain.

**Figure 4:**
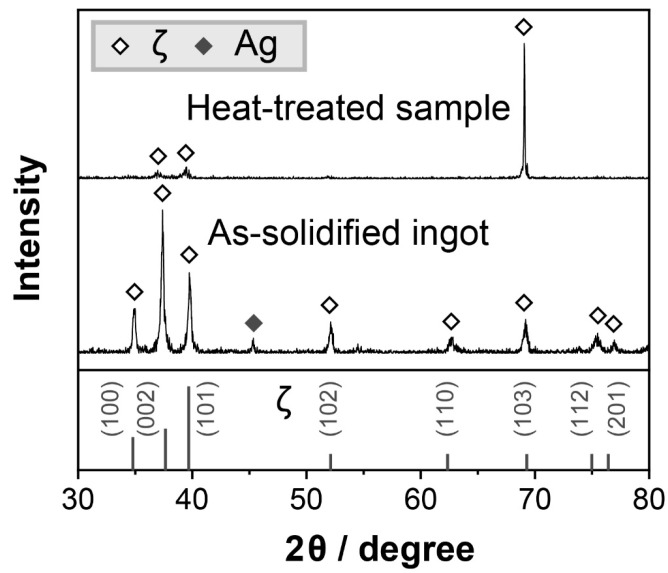
XRD results of the as-solidified ingot and heat-treated sample.

The compositions of spots 1 and 2, shown in Figure 2(a-2,b-2), respectively, are listed in Table 1 based on the EDS results. The XRD results obtained by scanning the entire surface of each sample are shown in Figure 4. The combined EDS and XRD confirm that each sample comprises the ζ (Ag_3_In) phase. No phase transition was found in the ζ (Ag_3_In) phase at a temperature range of room temperature to 520 °C. Although a small amount of Ag remained in the as-solidified ingot, it was eliminated during the subsequent heat treatment. Figure 4 shows that the distribution of peaks in the as-solidified ζ (Ag_3_In) ingot perfectly matches the reference powder diffraction file (PDF) data because of the presence of various small grains with random orientations resulting from rapid solidification. However, because the grains grew larger after the heat treatment, the grain orientation exhibited pronounced anisotropy along the (103) facet, suggesting a fast-growing orientation.

Based on these observations, a pure and densified ζ (Ag_3_In) phase bulk sample was successfully fabricated, providing the foundation for further thermomechanical measurements.

### 3.2. Hardness of ζ (Ag_3_In) Phase from 30 °C to 200 °C

**Figure 5:**
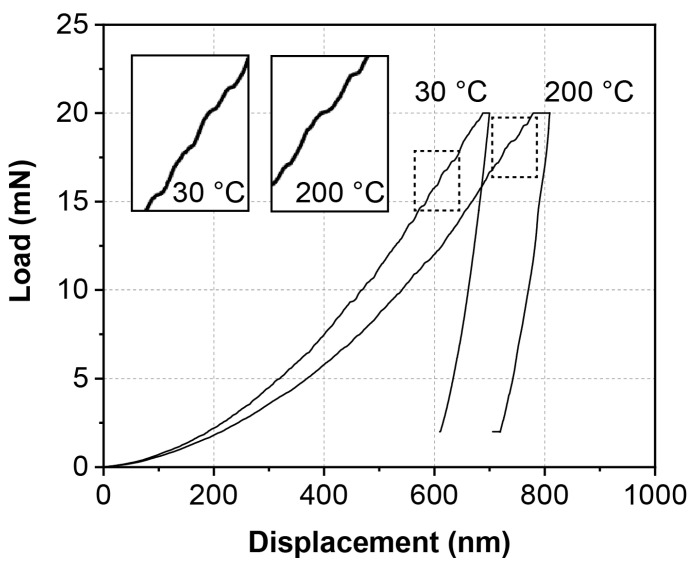
Load–displacement curves of ζ (Ag_3_In) phase at 30 °C and 200 °C.

Firstly, the displacement–load curves of the ζ (Ag_3_In) phase taken from nanoindentation tests at 30 °C and 200 °C are shown in Figure 5. “Pop in” phenomena are observed from both curves during loading, as shown from the magnified illustrations in Figure 5. This indicates that dislocations were activated under both temperatures [29]. Meanwhile, the “Pop in” phenomenon does not seem to increase at 200 °C, which, to some extent, suggests the good thermal stability of the ζ (Ag_3_In) phase. In addition, two characteristics of the ζ (Ag_3_In) phase can be deduced from the 200 °C curves. Firstly, no significant creep behavior is observed in the ζ (Ag_3_In) phase at 200 °C. Secondly, the ζ (Ag_3_In) phase does not shown significant softening at 200 °C.

**Figure 6:**
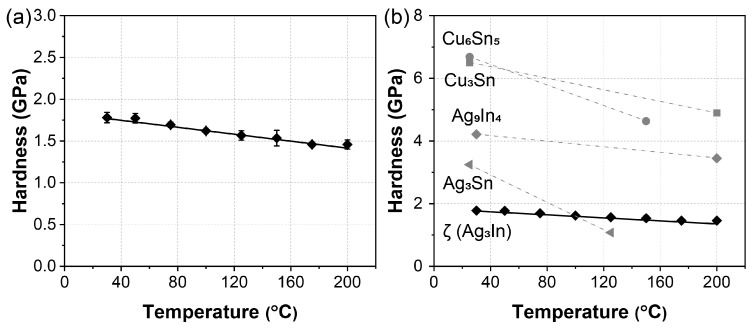
Hardness of IMCs at elevated temperatures. (**a**) Hardness of ζ (Ag_3_In) phase from 30 °C to 200 °C. (**b**) Hardness of Cu_6_Sn_5_ [31], Cu_3_Sn [32], Ni_3_Sn_4_ [33], Ag_3_Sn [34], Ag_9_In_4_ [26], and ζ (Ag_3_In) phase at elevated temperatures.

The hardness of the ζ (Ag_3_In) phase at elevated temperatures, ranging from 30 °C to 200 °C, is summarized in Figure 6a. Initially, at room temperature (30 °C), the ζ (Ag_3_In) phase exhibits a hardness of approximately 1.78 GPa. This is consistent with the hardness of the ζ (Ag_3_In) phase formed in the Ag-In TLP-bonded joint, as observed in our previous study [25]. As the temperature increases, the hardness decreases linearly, reaching 1.46 GPa at 200 °C. The relationship between the hardness (E) and temperature (T) can be described by the following equation [24]:

E = a − b × t,(1)

where a and b are constants with regressed values of 1.84 and 0.00216, respectively. A lower b values signifies a slower decline in hardness with increasing temperature, indicating better thermostability. This linear decline differs from the behavior observed in our previous study on Ag_9_In_4_, where the hardness remained nearly constant from 30 °C to 125 °C before exhibiting an exponential decrease up to 250 °C [26]. The hardness obtained from the nanoindentation tests is primarily associated with dislocation movement [30], which is influenced by thermally activated vacancies [29]. Based on the phase diagram shown in Figure 3, the melting point of the ζ (Ag_3_In) phase is approximately 660 °C, whereas gamma-Ag_9_In_4_ undergoes a solid-state reaction to the ζ (Ag_3_In) phase at around 300 °C. Consequently, this suggests that a temperature of 200 °C may not have been sufficient to activate thermal vacancies, thereby causing a noticeable softening effect in the ζ (Ag_3_In) phase. This phenomenon is consistent with the maintenance of hardness behavior observed in Ag_9_In_4_ within the lower temperature range of 30–125 °C.

**Figure 7:**
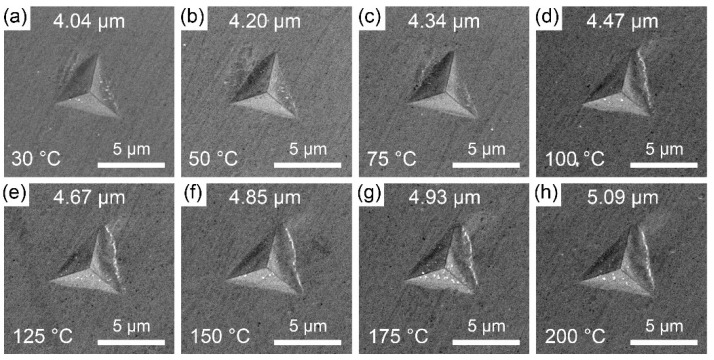
Indent images of ζ (Ag_3_In) phase obtained at (**a**) 30 °C, (**b**) 50 °C, (**c**) 75 °C, (**d**) 100 °C, (**e**) 125 °C, (**f**) 150 °C, (**g**) 175 °C, and (**h**) 200 °C.

The slight and linear decrease in the hardness of the ζ (Ag_3_In) phase was further confirmed through an analysis of the indent sizes, as shown in Figure 7. With increasing temperature, the size of the indenter slightly increases from approximately 4.04 μm at 30 °C to 5.09 μm at 200 °C. Notably, no cracks were observed at the tip or slip bands around the edge for any of the indents.

Figure 6b summarizes the hardness values of the various IMCs at elevated temperatures. At room temperature, the ζ (Ag_3_In) phase exhibited the lowest hardness among the IMCs. Meanwhile, as the temperature increased, the decrease in the hardness (as reflected by the b value) of the ζ (Ag_3_In) phase was also the smallest. This can be attributed in part to its high melting temperature. Consequently, these results indicate that the ζ (Ag_3_In) phase exhibits low hardness and superior thermostability in terms of its hardness behavior within a temperature range of 30–200 °C.

### 3.3. Young’s Modulus of ζ (Ag_3_In) Phase from 30 °C to 200 °C

**Figure 8:**
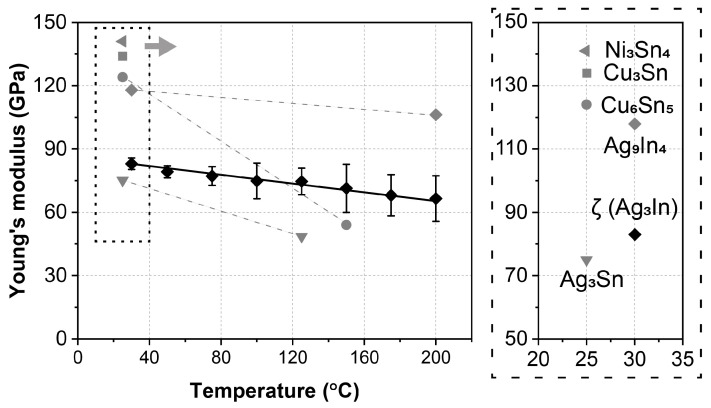
Young’s modulus of ζ (Ag_3_In) phase from 30 °C to 200 °C, with reference data for Cu_6_Sn_5_ [31], Cu_3_Sn [36], Ni_3_Sn_4_ [33], Ag_3_Sn [34], and Ag_9_In_4_ [26].

The Young’s modulus of the ζ (Ag_3_In) phase, at temperatures ranging from 30 °C to 200 °C, is summarized in Figure 8. The Young’s modulus of the other IMCs are also included. At room temperature (30 °C), the Young’s modulus is approximately 82.3 GPa. As the temperature increases, these values exhibit a linear decrease, reaching 66.5 GPa at 200 °C. This linearly decreasing trend aligns with the behavior observed in most IMCs and can be attributed to the elongation of the interatomic distances caused by volume expansion during heating [35]. The relationship between the Young’s modulus (E) and temperature (T) can be expressed as [24]:

E = c − d × t,(2)

where, c and d are constants, and the regressed values are 84.9 and 0.095, respectively.

Compared to other IMCs, as shown in Figure 8, several characteristics of the ζ (Ag_3_In) phase are revealed. First, at room temperature, the ζ (Ag_3_In) phase possesses a significantly lower Young’s modulus than Ni_3_Sn_4_, Cu_3_Sn, Cu_6_Sn_5_, and Cu_3_Sn but only slightly higher than that of Ag_3_Sn. Second, at elevated temperatures, the rate of decrease (as reflected by the d value) for the ζ (Ag_3_In) phase is slower than that of Cu_6_Sn_5_ and Ag_3_Sn but similar to that of Ag_9_In_4_. In summary, the ζ (Ag_3_In) phase has a low Young’s modulus with excellent thermostability compared to other IMCs in the temperature range of 30–200 °C.

### 3.4. CTE of ζ (Ag_3_In) Phase from 30 °C to 250 °C

**Figure 9:**
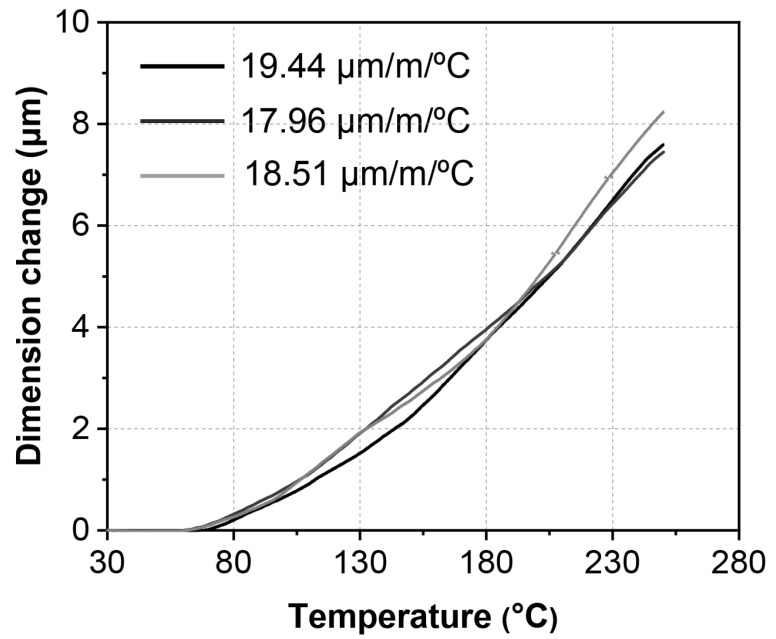
Dimensional change of ζ (Ag_3_In) phase from 30 °C to 250 °C.

Figure 9 shows the dimensional changes in the ζ (Ag_3_In) phase caused by thermal expansion during the heating. The dimensional changes of the ζ (Ag_3_In) phase during a temperature change from 30 °C to 70 °C cannot be recorded due to the cancellation effect resulting from the expansion of the glass bar used for detection and the tested specimen. Therefore, the total dimensional change is considered when heating the specimen from 70 °C to 250 °C, and this value is approximately 7.76 ± 0.35 μm, averaged by the three measurements, as illustrated in Figure 9. Consequently, the averaged CTE value is 18.63 ± 0.61 μm/m/°C. In comparison, the obtained CTE value of Ag_9_In_4_, as investigated in our previous study, was 20.6 μm/m/°C [26].

For comparison, the average CTE values of other IMCs are listed in Table 2. The CTE value of the ζ (Ag_3_In) phase closely resembles that of Cu_3_Sn, Cu_6_Sn_5_, and Ag_3_Sn, placing it at a medium level among the IMCs.

## 4. Discussion

Thermal stress, arising from the CTE mismatch among chips, the die-attach layer, and the substrate, is one of the primary causes of power module failure during operation [39]. However, modifying the established materials for chips and substrates is typically not feasible. Consequently, the die-attach joint plays a critical role in evaluating and potentially mitigating thermal stress to ensure reliable performance [35,36]. In other words, the thermomechanical properties of IMCs are crucial when TLP bonding is employed as a die attachment process. Generally, thermal stress primarily accumulates at the interface between the chip and IMC die-attach joint, rendering it the most vulnerable area to fracture. The approximate thermal stress at this interface on the IMC side (σ_I_), caused by temperature fluctuations, can be expressed as [10]:

σ_I_ = (α_I_ − α_C_) × (T_M_ − T_m_) × E_I_, (3)

where, α_I_ and α_C_ represent the CTE of the IMC and chip, respectively. T_M_ and T_m_ denote the maximum and minimum temperatures during operation, respectively. Thermal stress can be determined by the CTE difference between the chip and IMC, the temperature variation, and the Young’s modulus of the IMC itself. Therefore, a die-attach layer with a lower CTE and Young’s modulus is expected to reduce the level of thermal stress.

These findings indicate that the ζ (Ag_3_In) phase possesses one of the lowest Young’s moduli among other IMCs, while its CTE falls within a moderate range. These characteristics make the ζ (Ag_3_In) phase a superior alternative to Cu_6_Sn_5_, Cu_3_Sn, Ni_3_Sn_4_, and Ag_9_In_4_ when thermal stress is a key consideration. Additionally, the high melting temperature of the ζ (Ag_3_In) phase, along with its high thermostability, makes it appealing over other IMCs, including Ag_3_Sn. Furthermore, the ductility of the ζ (Ag_3_In) phase, as observed in our previous study, can help prevent brittle failure, which is common in other IMCs [25]. This ductility is crucial for the long-term reliability of die-attach joints.

In summary, among the currently used IMCs, the ζ (Ag_3_In) phase exhibits superior properties for serving as a die-attach joint in high-power modules. However, the high costs of the raw materials, Ag and In, as well as the lengthy bonding time (40 min at 250 °C [25]) required to form the ζ (Ag_3_In) phase, remain challenges impeding its broader industrial application.

## 5. Conclusions

In this study, pure and densified ζ (Ag_3_In) samples were successfully fabricated. The hardness, Young’s modulus, and CTE of these samples were investigated at elevated temperatures. The conclusions are summarized as follows:

(1) Pure and densified ζ (Ag_3_In) bulk samples were fabricated by being cast in a water-cooled steel mold, followed by undergoing heat treatment at 520 °C for 30 h. Both the as-cast and heat-treated samples comprised the ζ (Ag_3_In) phase, and no phase transition was found. The heat treatment process was used for homogenization.
(2) As the temperature increased from 30 °C to 200 °C, the hardness of the ζ (Ag_3_In) phase decreased linearly from 1.78 GPa to 1.46 GPa. Correspondingly, the Young’s modulus decreased linearly from 82.3 GPa to 66.5 GPa. Both values are lower than those of the other commonly used IMCs. Furthermore, the ζ (Ag_3_In) phase exhibited relatively good thermostability in terms of these properties.
(3) The average CTE of the ζ (Ag_3_In) phase over the temperature range of 70–250 °C was approximately 18.63 ± 0.61 μm/m/°C, which is similar to that of Cu_3_Sn, Cu_6_Sn_5_, and Ag_3_Sn, placing it at a moderate level among the other IMCs.

## Figures and Tables

**Table 1:** Compositions of EDS spots 1–3 in Figure 2.

	1	2
Ag (at. %)	75.86	76.07
In (at. %)	24.14	23.93
Estimated phase	ζ (Ag_3_In)	ζ (Ag_3_In)

**Table 2:** Average CTE of IMCs according to data in the literature.

	Ni_3_Sn_4_ [37]	Ag_3_Sn [38]	Cu_3_Sn [37]	Cu_6_Sn_5_ [37]	Ag_9_In_4_ [26]	ζ (Ag_3_In)
CTE (µm/m/°C)	13.7	18.1	19.0	16.3	20.6	18.6

## Data Availability

All data are included in this paper.
